# Boston Bombings and Resilience – What Do We Mean by This?

**DOI:** 10.3389/fpubh.2014.00025

**Published:** 2014-04-07

**Authors:** Dan Hanfling

**Affiliations:** ^1^Emergency Preparedness and Response, Inova Health System, Falls Church, VA, USA

**Keywords:** Boston bombings, resilience, response to marathon bombings, Peleg and Shenhar, security measures after Boston bombings

On Monday, April 15, 2013, several hours into the running of the world renowned Boston Marathon, two bombs exploded seconds apart at the crowded finish line. Three spectators died in the initial explosions, including an 8-year-old boy. Over 250 people were injured, many looking more like they were on the streets of Baghdad than in Boston. Single and double amputations, shrapnel wounds, and a sidewalk slick with bright red blood set the scene for one of the worst acts of terrorism on US soil since the 9–11 attacks. While not comparable to the magnitude of deaths and injuries inflicted on that terrible day in 2001, this type of “homegrown radicalized” terror has been thought to be a harbinger of events to come. And so, the issue of how our emergency response services can be expected to react and respond to similar events in the future is a topic of legitimate discussion. What is the proper balance to strike between ensuring security and imposing restrictions on civil society?

In their article, Peleg and Shenhar, our Israeli colleagues, question the decisions taken by emergency response authorities at the local, state, and Federal levels in response to the Boston Marathon bombings (1). They suggest that the law enforcement response may have been an over-reaction, resulting in greater harm than good. They note the near complete shutdown of the city of Boston, and the shelter in place order given to tens of thousands of residents, as incentives that demonstrate how easy it can be to disrupt civil society. The experiences of the Israeli civil and defense authorities provide important lessons for our own preparedness and response efforts. In this case, however, the arguments presented by the authors are not completely valid in describing the situation faced by Boston area local authorities responding to this event. These events are relatively rare occurrences in the United States. A show of force, and exertion of control, is not unexpected. Moreover, the continuous media cycle, with constant attention paid to the latest headline grabbing news, is a part of the culture. We have grown to accept such constant drumming – whether it is focused on terror, or that latest reports of bad weather.

With regards to the emergent management of the event, the authors conflate the responsibilities of local and regional response authorities with those of the US government in responding. For students of incident management and response, it is important to call out the distinctions between a national and a local and state response. The approximately 1000 National Guard present in Boston, were primarily used to secure the Marathon race route, and were not federalized (2). The city of Boston and its surrounding communities were not “secured by thousands of troops,” as the authors contend. The National Guard elements remained under the distinct authority of the Governor of Massachusetts. So, in fact, this was not really a “US Response,” but a local and state effort. By law and convention, all disasters are managed locally, with federal support, if required or requested. While there was a significant FBI response, they came in support of local law enforcement authorities.

The second point to be made relates to the notion of resilience – what it is and how are communities defined as “resilient”? The authors suggest that on account of the large scale dragnet imposed upon the city, and the closure of key transportation assets, the city’s core functions were “disrupted… on a massive scale.” Does disruption of service necessarily equate with lack of resilience? The citizens of Boston would probably argue otherwise. After all, their “Boston Strong” campaign, supported by the city’s two landmark sports franchises, the Boston Red Sox, and the Boston Bruins, helped to capture the sentiment of those affected by the attacks, and demonstrated the resolve to move ahead and begin the healing process (3). Moreover, from a health and medical response perspective, the actions and decisions taken by citizens and first responders at the scene of the bombings, and by medical staff assembled at Boston’s premier medical institutions, saved many lives and reduced the morbidity from injuries sustained by victims of the bombings. Life saving maneuvers were performed by men and women, in and out of uniform, lining the site of the race finish, without regard for personal safety. The application of tourniquets was highlighted as a critically important intervention, as was the rapid implementation of hospital emergency operations plans (4, 5).

In the ongoing evolution of the science of disaster medicine and crisis response, we will continue to use language and phrases that may mean different things to different audiences at different times. And as practitioners in the field, it is our responsibility to choose our language wisely. Like the notion of “surge capacity” before it, the use of the term “resilience” has become ubiquitous, and indeed may not always be relevant to the issues being discussed. In the Peleg and Shenhar article, the notion that the response to the bombings may have been harmful really does not take into account evidence of the many successes involved in the law enforcement, health, medical, and community responses that sprung into action.

In answering the question posed at the outset of this piece – what do we mean when we say “resilience” – Boston’s own Dr. Leonard Marcus, in his extensive work on the role leadership plays in emergency preparedness and response, notes the causality linking “bad leadership” to becoming a “public health risk factor” (6). If we agree with this assumption, then it stands to reason that good leadership builds resilience. So what we are really exploring here are the decisions taken by authorities responsible for the consequence management of the Boston Marathon bombings. Were the right response strategies put in place? Did the means justify the ends? It is evident that the law enforcement efforts resulted in the timely capture of the perpetrators. And the robust health and medical response, both on-scene and at Boston’s hospitals, limited the degree of suffering sustained by victims of the attack. In the United States, where these events thankfully remain infrequent occurrences, what lessons can be applied to future events? In examining the responses in Boston, can we say that there was effective leadership and management of civic duties such that restrictions on civil society were kept to a minimum, while the public’s confidence in returning to participate in Boston’s open, civic society were enhanced? It is apparent that the answer is a resounding “yes.”
